# Measurement of Dermal Ammonia Emission Using a Passive Flux Sampler and Its Association with Autonomic Nervous System Activity in Medical Workers: A Preliminary Study

**DOI:** 10.3390/s26113318

**Published:** 2026-05-23

**Authors:** Satomi Asai, Shiro Ikeda, Masaru Shiraiwa, Noboru Takanashi, Kazuo Umezawa, Kentaro Wakamatsu, Yoshika Sekine

**Affiliations:** 1 Department of Laboratory Medicine, Tokai University School of Medicine, Isehara 259-1193, Kanagawa, Japan; sa@tokai.ac.jp; 2 Gastec Corporation, Ayase 252-1195, Kanagawa, Japan; 3 Department of Clinical Laboratory Technology, Tokai University Hospital, Isehara 259-1193, Kanagawa, Japan; 4 Department of Emergency and Critical Care Medicine, Tokai University School of Medicine, Isehara 259-1193, Kanagawa, Japan; 5 Department of Respiratory Medicine, National Hospital Organization Omuta National Hospital, Omuta 837-0911, Fukuoka, Japan; 6 Department of Chemistry, School of Science, Tokai University, Hiratsuka 259-1292, Kanagawa, Japan

**Keywords:** skin gas, ammonia emission, passive flux sampler, heart rate variability, autonomic nervous system, non-invasive sensing, stress monitoring

## Abstract

**Highlights:**

**What are the main findings?**

- Dermal ammonia emission was associated with HRV indices related to autonomic nervous system activity, increasing under conditions characterized by lower HF and/or higher LF/HF, whereas elevated HF levels corresponded to lower ammonia emission (*r* = −0.47, *p* < 0.001).
- Temporal fluctuations in dermal ammonia emission were associated with changes in HRV indices under real-world working conditions.

**What are the implications of the main findings?**

- Dermal ammonia emission may have potential as a non-invasive approach for monitoring stress-related physiological responses.
- Non-invasive dermal gas sensing may contribute to future physiological monitoring in occupational settings.

**Abstract:**

Medical workers are frequently exposed to high-stress environments, highlighting the need for non-invasive stress monitoring strategies based on autonomic nervous system activity. Ammonia emitted from the human skin surface has been reported to increase under physical and psychological stress; however, its relationship with autonomic nervous system (ANS) dynamics remains unclear. In this study, we performed simultaneous, time-resolved measurements of dermal ammonia emission and heart rate variability (HRV) in 11 medical workers during 3 h of routine work. Dermal ammonia emission flux was continuously monitored using a passive flux sampler (PFS) coupled with ion chromatography, while autonomic nervous system activity was assessed by Holter electrocardiography. The temporal profiles of ammonia emission were analyzed in relation to HRV indices, including high frequency (HF) and the low-frequency-to-high-frequency ratio (LF/HF). Dermal ammonia emission increased under conditions characterized by lower HF and/or higher LF/HF, whereas elevated HF was associated with reduced ammonia emission (*r* = −0.47, *p* < 0.001). Furthermore, temporal fluctuations in ammonia emission were associated with sympathetic–parasympathetic switching. These findings suggest that dermal ammonia emission may be associated with HRV-related physiological responses under real-world working conditions and may have potential as a non-invasive indicator for stress-related physiological monitoring.

## 1. Introduction

Medical workers are at increased risk of burnout due to prolonged exposure to high-pressure environments [1,2]. In such settings, where excessive workload and stress are common, the balance of the autonomic nervous system (ANS) significantly influences both health status and performance. Therefore, stress management strategies, including ANS monitoring, are expected to yield promising outcomes. Stress responses have been evaluated using psychological, physiological, and biochemical approaches [3,4,5,6]. Questionnaires are widely used due to their simplicity; however, their subjectivity and limited reproducibility remain major concerns [4]. Physiological measurements, such as electroencephalography and blood pressure monitoring, provide objective insights but often require complex instrumentation and may impose a burden on participants [6]. Biochemical markers, including cortisol and salivary amylase, offer reliable indicators of stress; however, blood sampling is invasive, and saliva collection requires strict hygiene control [3,5]. These limitations highlight the need for alternative, non-invasive sensing approaches suitable for continuous monitoring in real-world environments. Therefore, we focused on trace gases emitted from the skin surface (skin gas) as a non-invasive and non-intrusive biological sample.

Human skin gas is a mixture of trace volatile compounds emitted from the body surface [7,8] and is perceived as body odour when it reaches the olfactory system. Ammonia is a typical component of human skin gas, and its pungent odour strongly contributes to body odour. When ammonia is produced in the body through internal metabolism, it diffuses from the blood through the dermal layers and/or is transported to the skin surface via sweating [8]. The amount of ammonia emitted through the dermis is known to increase under psychological stress and/or physical load [9,10,11], and the resulting body odour is often referred to as “fatigue odour” [9]. Previous studies have demonstrated that dermal ammonia emission increases under both psychological stress and physical load [9,10,11]. For example, stress induced by the Uchida–Kraepelin test increased ammonia emission across multiple body sites, particularly in regions associated with emotional sweating, such as the neck and palms [10]. In addition, physical activities such as walking and running have been reported to elevate ammonia and CO_2_ emission rates [11]. These findings suggest that dermal ammonia emission may reflect physiological states regulated by the ANS. Autonomic responses are commonly evaluated using heart rate variability (HRV), which reflects the balance between sympathetic and parasympathetic activity [12,13,14,15,16,17].

Despite its potential, the measurement of dermal gas emissions remains challenging due to their low concentrations and temporal variability. Conventional methods often require bulky instrumentation or lack sufficient time resolution for continuous monitoring under real-life conditions. In this context, passive flux samplers (PFS) offer a simple and non-invasive approach for quantifying dermal emission fluxes without interfering with natural physiological processes [8,9,10]. However, their applicability to dynamic physiological responses, such as ANS activity, has not been fully explored. Therefore, the aim of this preliminary study was to investigate the association between dermal ammonia emission and ANS-related HRV indices by simultaneously measuring skin-emitted ammonia using a passive flux sampling method and HRV under real-world working conditions. The present study explores the potential utility of combining dermal gas sensing with physiological monitoring as a non-invasive approach for stress-related assessment.

## 2. Methods

### 2.1. Participants

Simultaneous measurements of the dermal emission flux of ammonia and HRV were conducted by recruiting participants from the staff working at Tokai University School of Medicine Hospital. Although no formal medical screening or structured autonomic evaluation was performed, participants were recruited as apparently healthy volunteers based on self-reported health status and brief confirmation by the physician investigator (S.A.) at enrollment. Individuals with apparent illness or symptoms suggestive of conditions that could potentially affect autonomic nervous system activity were excluded from participation. The purpose of the study and the handling of personal information were explained to the participants, and 11 individuals (5 men and 6 women; aged 24–60 years, mean age: 48 years) who agreed to participate were selected. No specific exclusion criteria were established for the healthcare professionals. To minimize the influence of excessive perspiration, the test was conducted in air-conditioned hospital facilities. Participants were instructed to record their activities and work-related behaviours throughout the test period as thoroughly as possible for reference.

### 2.2. Measurement of Dermal Ammonia Emission

**Figure 1:**
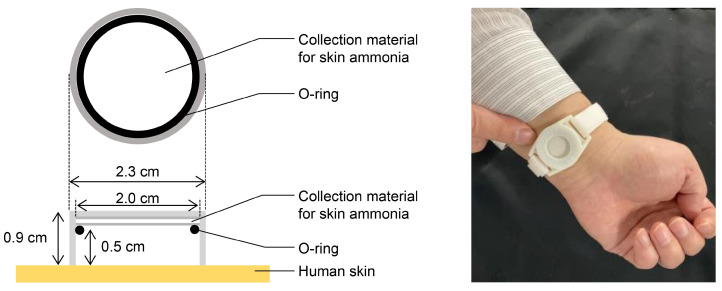
Schematic view of the passive flux sampler (PFS) used for the collection of ammonia emanating from the human skin surface. Note. The PFS was attached to the left wrist of participants using a wristband.

A passive flux sampler (PFS) [8,9,10] was attached to the participants’ left wrist to collect ammonia emitted from the skin surface. The collection time was set to 20 min, considering the sensitivity of the analysis, and the PFS was replaced with a new one at 20 min intervals. As described in detail in previous reports [9,18], the PFS comprises a main body, a collection material for gaseous ammonia, and an O-ring for retention (Figure 1). The collection material was prepared by impregnating cellulose filter paper (No. 51A, φ20 mm, 0.18 mm; Advantec Toyo, Ltd., Tokyo, Japan) with a solution of 2% phosphoric acid and 1% glycerine in methanol, followed by drying under reduced pressure in a vacuum desiccator for 1 h.

After collection, ammonium ions were extracted with 3.0 mL ultrapure water (Merck, Darmstadt, Germany, Milli-Q^®^ Type-1 ultrapure water system) and quantified using ion chromatography (IC). The IC system consisted of a pump (LC-20AD; Shimadzu Corporation, Kyoto, Japan), a conductivity detector (CDD-10AVP; Shimadzu Corporation, Kyoto, Japan), a column oven (CTO-10AVP; Shimadzu Corporation), and an auto-sampler (SIL-20A; Shimadzu Corporation). The following analytical conditions were used: column, 4.6 mm ID × 150 mm, Shim-pack IC-C4 (Shimadzu Corporation); eluent, 1.0 mM nitric acid with an isocratic mode; flow rate, 1.0 mL min^−1^; injection volume, 20 μL; and oven temperature, 40 °C. Dilution series of ammonium ion in Milli-Q water, 0.0, 0.20, 0.50, 2.0 and 5.0 mg L^−1^, were prepared from guaranteed reagent grade ammonium sulfate (FUJIFILM Wako Pure Chemical Corporation, Osaka, Japan) and used for calibration. All reagents except ammonium sulfate were purchased from Kanto Chemical Co., Ltd., Tokyo, Japan.

The dermal emission flux of ammonia *E* (ng cm^−2^ h^−1^) was calculated from the obtained ammonia collection amount *W* (ng), collection time *t* (h), and collection area *S* (=1.96 cm^2^) using Equation (1).

*E* = *W*/(*S*
*t*) (1)

The detection limit was defined as three times the standard deviation of the blank readings and was 10 ng cm^−2^ h^−1^. Because the present study was designed to evaluate dermal ammonia emission under real-world working conditions, no specific restrictions were imposed on participant diet or physical activity during the test, and the skin gas collection site was not subjected to any special cleaning prior to measurement.

### 2.3. Measurement of Heart Rate Variability

Participants were fitted with a Holter electrocardiogram (long-term electrocardiogram recorder RAC-3103; Nihon Kohden Corporation, Tokyo, Japan) that continuously recorded bipolar 2-lead electrocardiogram data at a sampling rate of 250 Hz for 3 h during work hours (9:00–12:00 or 13:00–16:00). HRV analysis was performed using consecutive 5 min segments of RR interval data, and each segment was analysed independently without overlap between adjacent segments. Frequency-domain analysis was performed on the obtained time-series data of RR intervals. The PFS was attached 5 or 10 min after the start of HRV measurement, and the corresponding 5 min HRV segments were aligned with the dermal emission flux of ammonia.

When sympathetic nervous system (SNS) activity increases in response to stressful stimuli, heart rate generally increases and RR intervals tend to shorten; however, heart rate alone does not fully reflect autonomic regulation, and such changes do not necessarily correspond directly to changes in autonomic balance. Therefore, HRV, which quantifies the temporal fluctuations in RR intervals, is widely used to assess ANS activity [12,13]. When RR interval time series are analysed using frequency-domain methods, the variability can be separated into low-frequency (LF) (0.04–0.15 Hz) and high-frequency (HF) (0.15–0.5 Hz) components. HF is primarily associated with respiratory-related vagal modulation and is commonly used as an index of parasympathetic nervous system (PNS) activity [14,15,16]. In contrast, LF reflects a combination of sympathetic and parasympathetic influences, including baroreflex-mediated regulation, rather than purely sympathetic activity. Therefore, the ratio of LF to HF (LF/HF) has been widely used as an index of sympathovagal balance; however, its physiological interpretation remains subject to ongoing discussion [17]. The LF/HF ratio and HF power were subsequently calculated using analytical software MemCalc/Chiram3 on the DSC-5500 system (Nihon Kohden Corporation).

### 2.4. Statistical Analyses

Calculation of statistics and graphing were performed using Microsoft^®^ Excel^®^ for Office 365 MSO (Version 2604, Build 16.0.19929.20172, 64bit, Microsoft Corp., Redmond, WA, USA). IBM SPSS Statistics 25 (IBM Corp., Armonk, NY, USA) was used to perform the statistical analyses. To investigate the relationships between dermal ammonia emission flux and HRV indices, Spearman’s rank correlation coefficients were calculated to evaluate associations between dermal emission flux of ammonia and HRV indices (HF and LF/HF), because the analysed variables did not satisfy the assumptions of normality and linearity required for Pearson’s correlation analysis. In addition, contingency table analysis was performed to further examine the relationship between dermal ammonia emission flux and LF/HF. Unless otherwise indicated, statistical significance was set at *p* < 0.05.

## 3. Results

**Figure 2:**
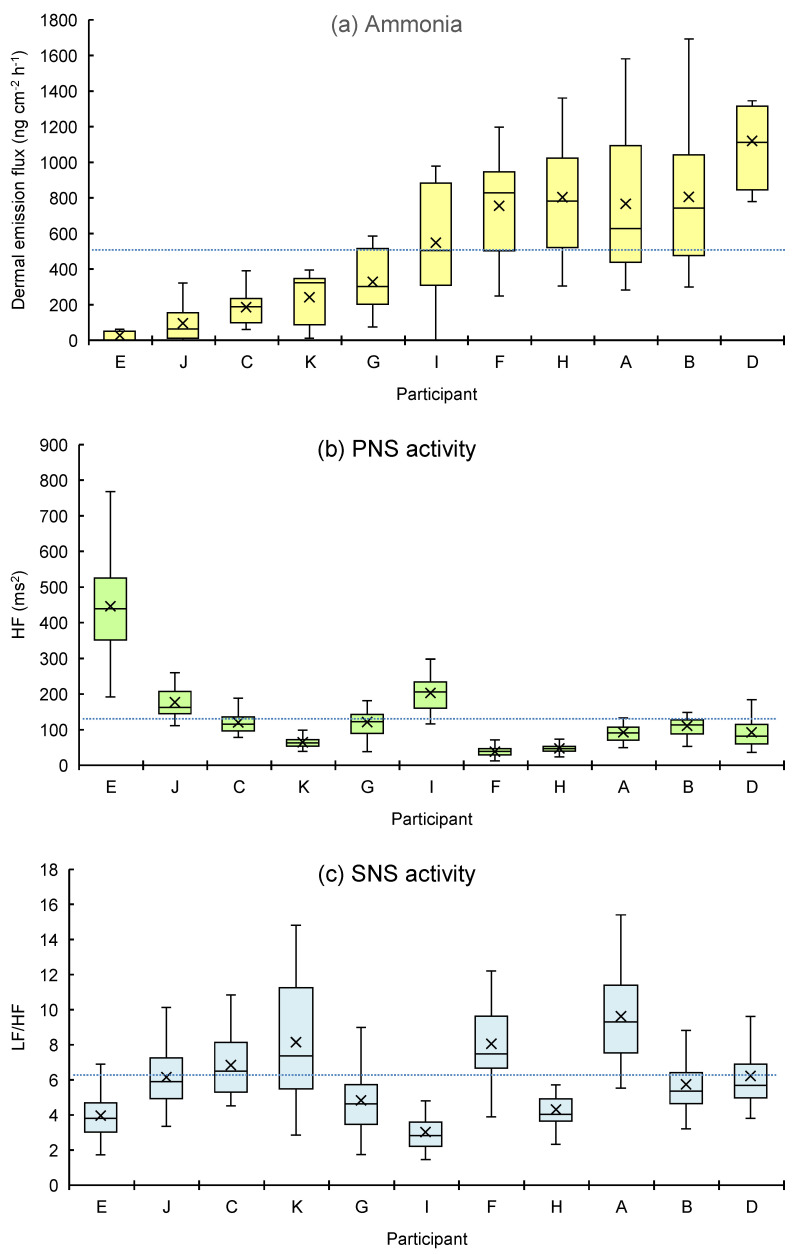
Overview of dermal ammonia emission flux and HRV indices across 11 participants. (**a**) Dermal emission flux of ammonia; (**b**) high-frequency (HF) power; (**c**) ratio of low frequency to high frequency (LF/HF). The symbol “×” indicates individual mean values. Participants are arranged in order based on their mean dermal ammonia emission levels. The dotted lines show the overall mean values.

The passive flux sampler enabled stable measurement of dermal ammonia emission under real-world working conditions, capturing both inter-individual differences and temporal variability. Figure 2 presents an inter-individual variation in the dermal emission flux of ammonia, high-frequency (HF) power, and the low-frequency to high-frequency (LF/HF) ratio measured in 11 participants (A–E). The symbol “×” indicates individual mean values, and participants are arranged in ascending order based on their mean ammonia emission levels. Substantial inter-individual variation in dermal ammonia emission flux was observed (Figure 2a). The lowest mean value was recorded for participant E (31 ± 44 ng cm^−2^ h^−1^), whereas the highest was observed for participant D (1120 ± 523 ng cm^−2^ h^−1^). Because of missing HRV data, 97 dermal ammonia emission datasets were available for comparison with the HRV indices. The overall mean ± standard deviation across the entire dataset was 518 ± 451 ng cm^−2^ h^−1^ (*n* = 97). Furukawa et al. [18] reported a dermal ammonia emission flux of 346 ± 234 ng cm^−2^ h^−1^ from the forearms of 10 healthy participants aged 21–23 years, indicating that the values observed in the medical workers tended to be relatively higher.

HF power and LF/HF ratio also exhibited marked inter-individual variation (Figure 2b,c). Participants with higher ammonia levels tended to exhibit lower HF values and higher LF/HF ratios, with this trend being particularly pronounced among participants E through K. However, the participant-averaged dermal ammonia emission does not appear to have a simple proportional relationship with these HRV indices.

**Figure 3:**
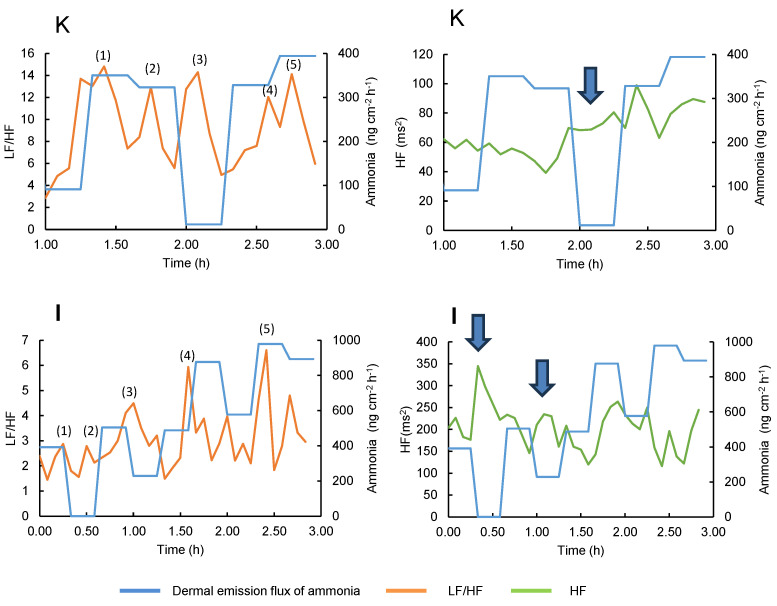
Representative time-course profiles of dermal ammonia emission flux and HRV indices in two participants (K and I). (**Left panels**) show ammonia emission flux and LF/HF ratio; (**Right panels**) show ammonia emission flux and HF power. Numbers in parentheses indicate representative changes in LF/HF, and the downward arrows indicate time points at which increases in HF were observed. Numbers (1)—(5) show distinct peaks in the LF/HF ratio.

To further explore the influence of ANS balance on ammonia levels, the temporal changes in dermal emission flux of ammonia, HF, and LF/HF were examined for each individual participant. All raw data are illustrated in Supplementary Figure S1. Across participants, dermal ammonia emission exhibited clear temporal variability over the 3 h monitoring period. Changes in ammonia emission were temporally associated with variations in HRV indices. In several cases, increases in LF/HF were followed by increases in ammonia emission, whereas simultaneous increases in HF were associated with attenuation or delay of ammonia elevation. Representative time-course data from participants K and I are shown in Figure 3.

In participant K, five distinct peaks in the LF/HF ratio (peaks 1–5) were observed at 1.25–2.75 h after the start of measurement. Except for peak (3), each LF/HF peak was followed by an increase in ammonia emission flux. In the case of peak (3), however, a simultaneous increase in HF was observed (↓), and a clear increase in ammonia emission was not observed at the corresponding time point. This observation may indicate that temporal changes in dermal ammonia emission are associated with fluctuations in autonomic balance rather than with a single HRV parameter alone. In other words, fluctuations in ammonia emission were temporally associated with sympathetic–parasympathetic switching (autonomic switching).

A similar trend was observed in participant I, who exhibited relatively low levels of psychological tension. Ammonia emission flux tended to increase with increases in the LF/HF ratio. However, at approximately 0.3 h (peak 1) and 1.0 h (peak 3), increases in HF were also noted, and no sharp increase in ammonia levels was detected at those times.

These observations suggest that temporal fluctuations in dermal ammonia emission may be associated with short-term changes in ANS-related HRV indices and reflect the balance between sympathetic and parasympathetic influences, rather than a single HRV index alone.

**Figure 4:**
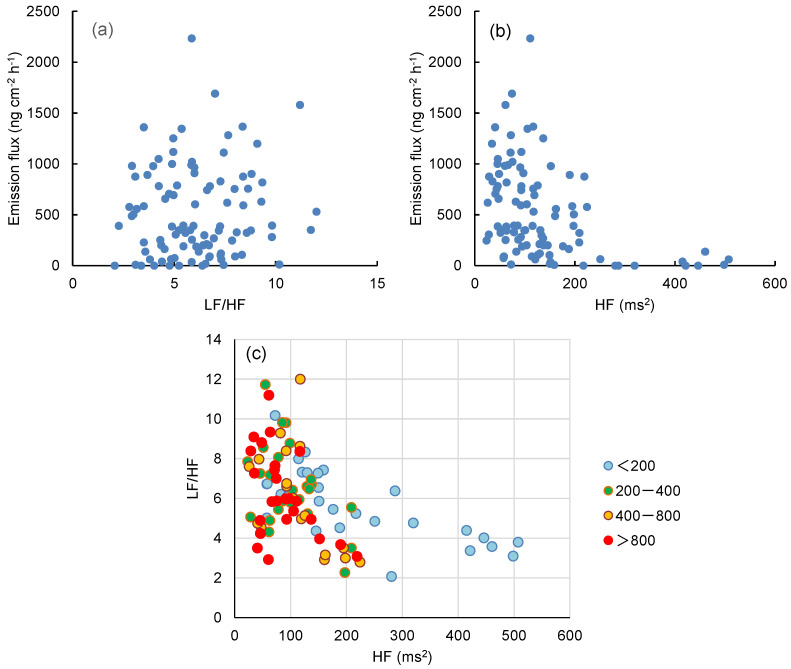
Relationships between dermal ammonia emission flux and both LF/HF and HF using the entire dataset (*n* = 97). (**a**) Dermal emission flux of ammonia versus LF/HF, (**b**) Dermal emission flux of ammonia versus HF power, and (**c**) Two-dimensional visualization of dermal ammonia emission as a function of LF/HF ratio and HF power. Colour represents ammonia emission flux.

Figure 4 shows the relationships between dermal ammonia emission flux and both LF/HF and HF using the entire dataset. A moderate negative correlation was observed between ammonia emission flux and HF (Spearman’s *r* = −0.471, *p* < 0.001), whereas no significant correlation was found between ammonia emission and LF/HF. Consistently, contingency table analysis did not provide evidence of a clear association between ammonia emission and LF/HF. To further examine these relationships, a two-dimensional visualization was constructed, as shown in Figure 4c. The plots were colour-coded into four levels according to dermal ammonia emission. The distribution of the plots indicates that ammonia emission varies across combinations of HF and LF/HF values. Notably, lower ammonia emission levels were frequently observed under conditions of higher HF.

Taken together, these results suggest that dermal ammonia emission tended to increase under conditions characterized by lower HF and/or higher LF/HF, whereas elevated HF was associated with lower ammonia emission levels. In addition, continuous monitoring demonstrated temporal fluctuations in dermal ammonia emission in parallel with changes in HRV indices during routine work.

## 4. Discussion

In this study, the dermal emission flux of ammonia and HRV were simultaneously measured in 11 medical workers. Medical workers are continuously exposed to psychologically and physically demanding environments, making non-invasive approaches for stress-related ANS monitoring particularly important. In this context, the present findings support the feasibility of dermal ammonia sensing as a practical and non-intrusive method for evaluating physiological responses under real-world working conditions. The present findings also support the potential utility of skin ammonia as a biomarker reflecting ANS responses in relation to HRV.

As shown in Figure 3, the time series data of the LF/HF and HF showed that the ANS responses of participants changed constantly. The participants were engaged in various tasks during the measurement period, including clinical testing, report writing, and communication with doctors, nurses, and patients. It is believed that their ANS responses to these tasks affected their HRV. Meanwhile, the dermal emission flux of ammonia also changed with time and was almost synchronized with the time course of the LF/HF and HF; Increases in LF/HF were followed by increases in ammonia emission, whereas simultaneous increases in HF were associated with attenuation or delay of ammonia elevation. These results suggest that increased dermal ammonia emission may occur under sympathetic-dominant conditions, whereas higher HF levels were associated with lower dermal ammonia emission in the participants examined in this study.

In this study, HF and LF/HF were selected as representative HRV indices because they are widely used as markers associated with parasympathetic activity and sympathovagal balance. Although additional parameters such as total power and normalized indices (nLF and nHF) may provide complementary information, these were not included in order to maintain a focused analysis of the primary outcomes. Time-domain parameters were not included because the present analysis focused on frequency-domain characteristics of ANS activity. We also observed no sex differences in the skin ammonia emission, and no effect of age or working hours. Therefore, such non-invasive measurement of ammonia is useful for stress management in medical workers, especially for monitoring ANS functions. Importantly, the present approach enables non-invasive and continuous monitoring of physiological responses under real-world working conditions, which is difficult to achieve using conventional biochemical or physiological measurements. This highlights the potential of dermal gas sensing as a practical tool for real-time stress assessment. However, the PFS used in this study has a limitation in that measurement results cannot be obtained on-site in real time. Nevertheless, recent advances in wearable technologies have enabled real-time sensing of trace levels of various skin gases [9,19,20,21]. Therefore, the findings of this study are expected to contribute to the further development of such sensor technologies.

Previous human studies have demonstrated correlations between blood ammonia concentrations and dermal ammonia emission [18,22], which provided the physiological rationale for focusing on dermal ammonia emission in the present exploratory study. SNS activity may indirectly contribute to elevated blood ammonia levels. One possible mechanism is muscle metabolism during stress or exercise. SNS activation enhances muscle activity and increases ATP-dependent energy demand, thereby accelerating amino acid catabolism and purine nucleotide turnover—both of which produce ammonia as a byproduct. Ogasawara et al. [23] demonstrated that during graded exercise, blood ammonia and noradrenaline levels rise in parallel, suggesting a connection between muscle sympathetic activity and ammonia production. Another plausible mechanism is reduced hepatic clearance. SNS dominance often suppresses parasympathetic tone, leading to decreased hepatic blood flow [24]. Reduced liver perfusion may impair the ornithine cycle, the primary pathway for converting ammonia into urea. Consequently, transient elevations in blood ammonia may occur, particularly under conditions such as sleep deprivation or chronic stress, which are common experiences among medical workers. Therefore, changes in HRV indices associated with autonomic regulation may be related to increased dermal ammonia emission in the present participants, although the underlying mechanisms remain unclear. These physiological mechanisms support the feasibility of using dermal ammonia emission as an indirect sensing signal reflecting systemic metabolic and autonomic responses. To the best of our knowledge, few studies have directly examined the relationship between ANS activity and dermal ammonia emission under real-world occupational conditions. Therefore, the present findings may provide novel insights into the feasibility of dermal ammonia sensing as a non-invasive indicator of autonomic responses.

This study has a potential limitation regarding participant selection and analytical sensitivity. All participants were medical professionals working in hospital settings, and due to the inherently demanding nature of their work, they are likely to be at increased risk for mental health issues [1,2]. Participants were recruited as healthy volunteers based on self-reported health status, and their health status was confirmed by the physician investigator at enrollment. However, no formal medical screening, laboratory evaluation, or strict exclusion criteria were applied. Therefore, the potential influence of latent or subclinical conditions affecting autonomic nervous system activity could not be completely excluded. Future studies with more rigorous screening criteria are warranted. In addition, HRV measurements were conducted at different times of day depending on participants’ work schedules. As ANS activity is known to exhibit diurnal variation, differences in recording time may have influenced baseline HRV levels. However, this study focused on temporal fluctuations within individuals rather than absolute comparisons between participants. Future studies under more controlled timing conditions are warranted. Therefore, to strengthen the findings on how ANS activity influences the dynamics of ammonia emission in humans, further studies involving a broader range of participants and a larger sample size are necessary. Additionally, measurement of dermal ammonia using the PFS method was conducted over a 20 min period, taking analytical sensitivity into account. However, since autonomic switching can occur on a second-by-second basis, it is difficult to capture these rapid transitions using the present measurement protocol. Future research should focus on the development of high-sensitivity, real-time dermal gas sensors with improved temporal resolution, enabling second-scale monitoring of autonomic dynamics. In addition, external factors such as sweating, skin temperature, and environmental conditions may influence dermal ammonia emission and should be carefully controlled or compensated for in future sensor development.

## 5. Conclusions

Recent studies have suggested that physical and psychological stress can induce dermal ammonia emission. In this study, simultaneous measurements of dermal ammonia emission and HRV suggested associations between ammonia emission and HRV indices related to autonomic nervous system activity. Specifically, dermal ammonia emission tended to increase under conditions characterized by lower HF and/or higher LF/HF, whereas higher HF levels were associated with lower ammonia emission levels. Moreover, temporal fluctuations in dermal ammonia emission were associated with changes in HRV indices. These findings suggest that dermal ammonia emission may be associated with HRV-related physiological responses in the participants examined in this study and may have potential as a non-invasive physiological monitoring approach. Further studies with larger and more diverse populations are warranted to validate these findings.

## Data Availability

All data gathered or analyzed during this study are included in this published article.

## Supplementary Materials

The following supporting information can be downloaded at: https://www.mdpi.com/article/10.3390/s26113318/s1, Figure S1: Results of the time-series measurement for LF/HF, HF, and dermal emission flux of ammonia for the 11 medical workers (A–K) while working at a hospital for 3 h.

## Abbreviations

The following abbreviations are used in this manuscript:

ANS	Autonomic nervous system
HRV	Heart rate variability
HF	High frequency
LF	Low frequency
SNS	Sympathetic nervous system
PNS	Parasympathetic nervous system
PFS	Passive flux sampler
