# Supplementary material for: Measurement of Dermal Ammonia Emission Using a Passive Flux Sampler and Its Association with Autonomic Nervous System Activity in Medical Workers: A Preliminary Study
Source: Sensors (Basel). 2026 May 23;26(11):3318. doi: 10.3390/s26113318 (PMC13259317; doi:10.3390/s26113318)
Supplement: Supplementary file 1 [file sensors-26-03318-s001.zip › sensors-4278608-supplementary.pdf]

S1 Fig

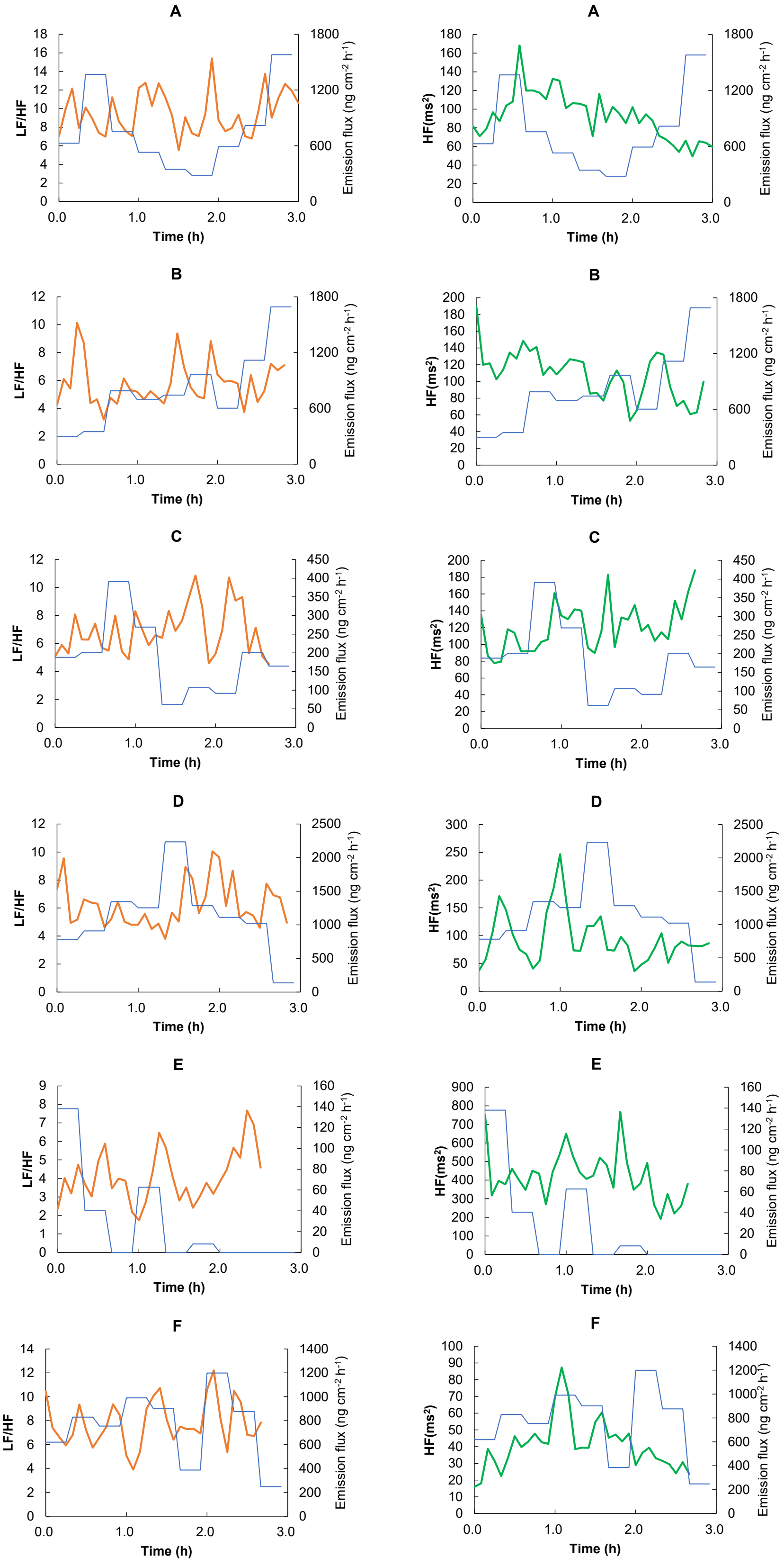

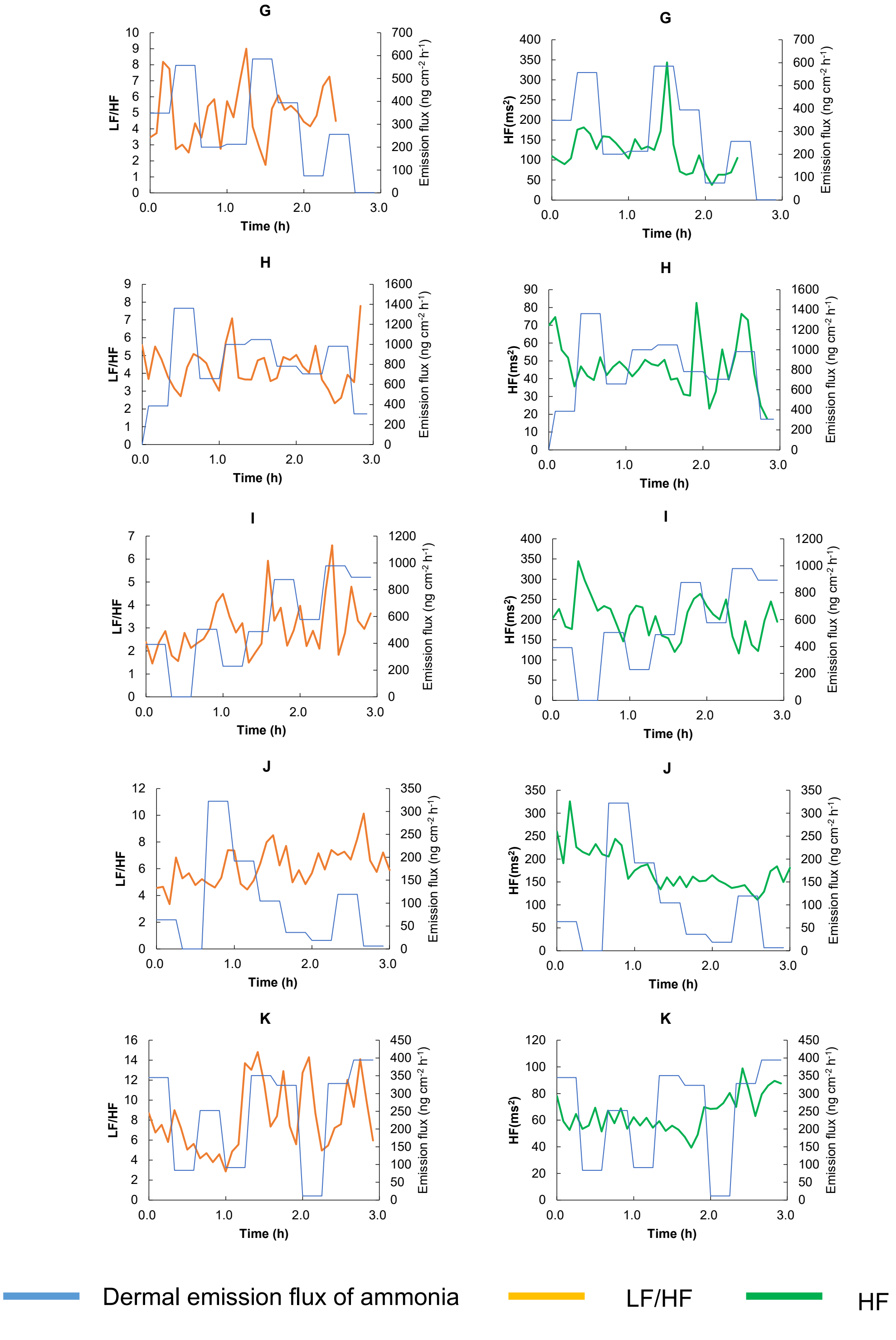

Figure S1. Results of the time-series measurement for LF/HF, HF, and dermal emission flux of ammonia for the 11 medical workers (A–K) while working at a hospital for 3 hours.
